# FcγR-Binding Is an Important Functional Attribute for Immune Checkpoint Antibodies in Cancer Immunotherapy

**DOI:** 10.3389/fimmu.2019.00292

**Published:** 2019-02-26

**Authors:** Xin Chen, Xiaomin Song, Kang Li, Tong Zhang

**Affiliations:** BeiGene (Beijing) Co., Ltd., Beijing, China

**Keywords:** FcγR, checkpoint blockade, antibody therapy, cancer immunotherapy, IgG isotype

## Abstract

T cells play critical roles in anti-tumor immunity. Up-regulation of immune checkpoint molecules (PD-1, PD-L1, CTLA-4, TIM-3, Lag-3, TIGIT, CD73, VISTA, B7-H3) in the tumor microenvironment is an important mechanism that restrains effector T cells from the anti-tumor activity. To date, immune checkpoint antibodies have demonstrated significant clinical benefits for cancer patients treated with mono- or combination immunotherapies. However, many tumors do not respond to the treatment well, and merely blocking the immune suppression pathways by checkpoint-regulatory antibodies may not render optimal tumor growth inhibition. Binding of the antibody Fc-hinge region to Fc gamma receptors (FcγRs) has been shown to exert a profound impact on antibody function and *in vivo* efficacy. Investigation of immune checkpoint antibodies regarding their effector functions and impact on therapeutic efficacy has gained more attention in recent years. In this review, we discuss Fc variants of antibodies against immune checkpoint targets and the potential mechanisms of how FcγR-binding could influence the anti-tumor activity of these antibodies.

## Introduction

Immune checkpoints refer to multiple inhibitory pathways that control the immune system to maintain self-tolerance and modulate the intensity of physiological immune responses in order to minimize pathological damage (1–3). Antagonizing antibodies against immune checkpoint inhibitory molecules has achieved great success in cancer treatment (1, 2). However, many tumors do not respond to the treatment, and antibody optimization (especially in the isotype selection) is essential for improving outcomes (4, 5). target-binding specificity, imparted by the antibody's variable region, is well-known to be critical for the primary functional activities of the antibody. However, mounting evidence has shown that the antibody's constant region also plays a crucial role, much of which is mediated through interaction of the crystallizable fragment (Fc) with Fcγ receptors (FcγRs) (6). Fc endows IgG antibodies with effector functions, which include antibody dependent-cellular cytotoxicity (ADCC), complement-dependent cytotoxicity (CDC), antibody-dependent cellular phagocytosis (ADCP), Induction of cytokines/chemokines and endocytosis of opsonized targets (7).

To date, therapeutic IgG antibodies (either approved or in clinical development) belong to the IgG1, IgG2 or IgG4 subclasses. Each IgG isotype has a distinct binding affinity to the various FcγRs, which are expressed differently on immune cells. A combination of these features leads to diverse and highly regulated antibody responses.

Antagonizing antibodies against major T-cell inhibitory pathways, such as PD-1/PD-L1 and CTLA-4, have become important parts of cancer therapeutics (1). Consequently, the next wave of therapeutic antibodies targeting alternative immunosuppression pathways (e.g., LAG-3, TIM-3, B7-H3, VISTA, CD73) are rapidly emerging (8). The majority of the immune checkpoint antibodies have low or significantly reduced binding to FcγRs to avoid potential ADCC and CDC, especially when the target molecule is expressed on effector T cells (9). However, for targets such as CTLA-4, TIGIT, and VISTA, competent Fc is required for optimal anti-tumor immune responses in various mouse models (10–12). The mechanisms of action (MOA) may involve the killing of regulatory T cells (Tregs), promoting immune synapse formation and production of pro-inflammatory cytokines due to cross-linking of FcγRs with the competent Fc.

In this article, we summarize the major properties of different IgG isotypes and FcγRs, describe the MOA of different immune checkpoint targets in inhibiting anti-tumor immunity and review the recent studies on the important roles of either binding or not binding to FcγRs in immune checkpoint antibody therapy. It should be noted that many of the findings come from mouse models; the clinical significance of these findings has yet to be determined.

## IgG Isotypes and FcγRs

In humans, there are four isotypes of IgG (IgG1-4), differing from the other in their binding profiles to various FcγRs and to complement subunits, such as C1q. IgG1 has the highest affinity to all FcγRs and C1q, leading to significant effector functions, such as ADCC, ADCP, and CDC (5, 13). Although human IgG3 can also mediate competent effector functions, it has a very long hinge region and complex disulfide bonds, resulting in significantly greater polymorphism, which may increase the risk of immunogenicity. Therefore, the IgG3 isotype is rarely chosen in antibody therapeutics (14) and is not further discussed in this review. In comparison, IgG2 and IgG4 induce significantly weaker or no ADCC and CDC (13). The binding features of different IgG isotypes to various FcγRs are summarized in Table 1 and discussed below.

**Table 1:** Binding activities of human FcγR to IgG isotypes and resulting effector functions.

**FcγR**	**Variants**	**IgG1**		**IgG2**		**IgG4**	
		**Affinity^a^**	**Effector functions**	**Affinity**	**Effector functions**	**Affinity**	**Effector functions**
I	NA	High	ADCP	None	None	High	ADCP, Cytokine release
IIa	H_131_	Medium	ADCP	Medium	Myeloid cell-induced ADCC^b^	Low	Receptorclustering^c^
	R_131_	Low		Low		Low	
IIb	I_232_^d^	Low	Clearance of IC, Immuno-suppression	None	None	Low	Clearance of IC, Immuno-suppression
	T_232_^d^						
IIIa	V_158_	Medium	ADCC	Low	None	Low	None
	F_158_	Low		None		None	

a *Affinity values are based on IC binding to FcγR, adapted from Bruhns et al. (13)*.

b *Based on Arce Vargas et al. (15)*.

c *Based on Oberst et al. (16)*.

d *The T_232_ variant is less potent in inhibitory activity than the I_232_ variant (17). However, the I_232_T mutation leads to significantly better phagocytosis (18)*.

The overall structures of IgG1, IgG2, and IgG4 are very similar with more than 90% sequence homology. The major differences reside in the hinge region and CH2 domain, which form primary binding sites to FcγRs (19–21). The hinge region also functions as a flexible linker between the Fab and Fc portion.

In addition to differential binding affinity to FcγRs, IgG4, and IgG2 demonstrate other unique features. IgG4 has a unique S_228_ in the hinge region, which allows for interchangeable disulfide bond configurations and formation of “half-antibodies” (22). *In vivo*, IgG4 with different specificity may shuffle, resulting in monovalent-bispecific antibodies (a process called “Fab-arm exchange”) (23). S_228_P mutation of IgG4 can efficiently eliminate fab-arm change. Therefore, the majority of recently approved therapeutic IgG4 antibodies adopt an S_228_P mutation (24). In IgG2, several disulfide bond isomers (IgG2A, IgG2B, and IgG2A/B) can be formed (25, 26). Many factors such as cell culture conditions or thermal stress contribute to the formation and equilibrium of different isomers (27). *In vivo*, IgG2A isomer can convert to the form of IgG2B (28). Among the three isomers, IgG2B has the most compact structure (26). In addition, as compared to the form of IgG2A, the IgG2B conformation imparts super-agonistic properties to immunostimulatory antibodies, such as anti-CD40 antibodies (29). The feature of IgG2 isomer transformation is FcγR-independent and its activity has been demonstrated for IgG2 CD40 mAb in the clinical trial CP870-893 (29).

In mice, IgG2A functionally resembles human IgG1, whereas mouse IgG1 is considered the closest functional equivalent of human IgG4. The D_265_A mutation can further reduce the affinity of mouse IgG1 for the Fc receptor, leading to a “silent Fc” and antibodies harboring this mutation have been widely used in mouse models to evaluate the effects of FcγR-binding on *in vivo* therapeutic efficacy (30–32).

Based on the differences in structure, function, and affinity for IgG binding, FcγRs are classified into three major groups: FcγRI, FcγRII (FcγRIIa and FcγRIIb) and FcγRIII (FcγRIIIa and FcγRIIIb) (13). Among them, FcγRI, FcγRIIa, and FcγRIIIa are activating receptors containing the signal transduction motif, immunoreceptor tyrosine-based activation motif (ITAM), in the γ subunit of FcγRI and FcγRIIIa, or in the cytoplasmic tail of FcγRIIa (14). In contrast, FcγRIIb is an inhibitory receptor. Cross-linking of FcγRIIb leads to the phosphorylation of the immunoreceptor tyrosine-based inhibitory motif (ITIM) and inhibitory signaling transduction (33).

## FcγRI

FcγRI is a high-affinity Fc receptor for both the monomeric IgG and immune complex (IC) (13). The affinities of FcγRI to IgG1 or IgG4 are similar (*K*_*D*_ of 1–10 nM*)*. In contrast, FcγRI has no binding to IgG2. FcγRI is mainly expressed on monocytes/macrophages, dendritic cells (DCs), and activated neutrophils. One of the major functions of FcγRI is to activate myeloid cells to phagocytose IgG1 and IgG-bound target cells *via* ADCP (34). Due to high-affinity binding of FcγRI to monomeric IgG and high serum concentrations of IgG (~15 mg/mL), it is believed that most FcγRI is occupied by endogenous IgG (35). However, a recent study has shown that stimulation of myeloid cells with cytokines, such as tumor necrosis factor-α (TNF-α) and interferon-γ (IFN-γ), could induce the clustering of FcγRI and increase the binding of FcγRI to ICs (36). Multiple studies have also shown that FcγRI plays an important role in modulating immune responses in autoimmune diseases, inflammation, and antibody therapy (37–39).

## FcγRIIa and FcγRIIIa

Both FcγRIIa and FcγRIIIa are low-affinity FcγRs, which bind weakly to monomeric IgG, but strongly to IC. FcγRIIa and FcγRIIIa receptors are primarily expressed on monocytes/macrophages, dendritic cells, natural killer cells and platelets. FcγR polymorphisms exist in FcγRIIa and FcγRIIIa receptors, resulting in two isoforms of each receptor: H_131_ and R_131_ of FcγRIIa(40), V_158_ and F_158_ of FcγRIIIa (41), respectively. FcγRIIa-H_131_ variant is considered a high responder as compared to R_131_ variant (low responder) due to a higher affinity for IgG1 and increased effector functions (such as phagocytosis) (13, 22). Similar to FcγRI, FcγRIIa is one of the major phagocytic FcγRs that mediates ADCP. In human, FcγRIIIa is the primary receptor for NK- and macrophage-mediated ADCC. FcγRIIIa-V_158_ variant (high responder) has a higher affinity for IgG1 and can also interact with IgG4 (13). Functionally, IgG-induced NK cell activity is increased in FcγRIIIA-V/V_158_ homozygotes compared with FcγRIIIA-F/F_158_ individuals (42).

## FcγRIIb

FcγRIIb is expressed on many types of immune cells including B cells, DCs, monocytes/macrophages, mast cells and basophils (33). In addition, FcγRIIb was found to be expressed on liver sinusoidal endothelial cells (LSEC) and plays an important role in IC clearance (43). On B cells, FcγRIIb functions as a primary inhibitory FcγR to suppress B cell activation and antigen internalization after binding to the immune complex (33). FcγRIIb also inhibits the type I interferon production by DCs. The binding affinities of monomeric IgG to FcγRIIb are extremely low (*K*_*A*_ ≈ 2 x 10^5^M^−1^), whereas the affinities of IC to FcγRIIb are significantly higher (13). Despite the critical roles of FcγRIIb in the negative regulation of immune responses, several studies have shown that FcγRIIb is required for the induction of efficient anti-tumor activity by agonistic anti-TNF receptor superfamily-antibody therapeutics such as anti-CD40 antibodies (44, 45). The overall binding features of human FcγR to IgG isotypes are summarized in Table 1.

## Mouse FcγRIV

In addition to the FcγRs described above, in mice, there is a unique FcγR (i.e., FcγRIV), whose expression is restricted to myeloid lineage cells (46). FcγRIV bind to mouse IgG2a and IgG2b with intermediate affinity and plays critical roles in IgG2a- and IgG2b-mediated *in vivo* efficacy (46, 47). Mouse FcγRIV is functionally similar to human FcγRIIIa, but not expressed on natural killer cells (47). In a mouse model, anti-CTLA-4 antibody-mediated depletion of Tregs is largely dependent on FcγRIV (10).

## Fc engineering to reduce or eliminate FcγR binding

Several modifications to IgG can directly affect their binding to FcγRs. The N_297_A mutation was the first mutation to be described with significantly reduced FcγR-binding (48). It was later demonstrated that mutations of residues 234 and 235 in the lower hinge region (EU numbering system) to alanine could also lead to significantly reduced FcγR-binding; the L_234_A/L_235_A double mutation on the human IgG1 backbone is also known as the “LALA” mutation (49). In addition, hybrid antibody isotype IgG2m4, which is based on the IgG2 with four key amino acid residue changes derived from IgG4 (H_268_Q, V_309_L, A_330_S, and P_331_S), has been shown to have significantly reduced FcγR binding (50).

## Immune Checkpoint Molecules and Their Therapeutic Antibodies

### CTLA-4

CTLA-4 (cytotoxic T-lymphocyte-4, or CD152) is a member of the Ig superfamily, which plays a critical role in inhibiting T-cell immunity (51). The ligands are the B7 family members, CD80 (B7-1) and CD86 (B7-2). As a CTLA-4-related protein, CD28 is constitutively expressed on naïve T cells and enhances T-cell activation when engaged by B7-1/2 on antigen-presenting cells (APC) (52, 53). In contrast, CTLA-4 surface expression increases in a day or two after T cell activation (51, 52). CTLA-4 is also highly expressed on Tregs and plays an important role in the homeostasis and suppressive functions of Tregs (54). There is no known canonical immunoreceptor tyrosine-based inhibitory (ITIM) motif in the cytoplasmic tail of CTLA-4 (55). The exact signaling pathway of CTLA-4 upon engagement with its ligands still remains largely unknown. Accumulating evidence suggested that CTLA-4 primarily exerted its inhibitory functions by competing off CD28 binding to CD80 and/or CD86, due to the higher affinity of CTLA4 to CD80 or CD86 (55). In addition, CTLA-4 has been shown to down-regulate CD80 and CD86 on APC, thus inhibiting CD28-mediated co-stimulation (54).

**Figure 1:**
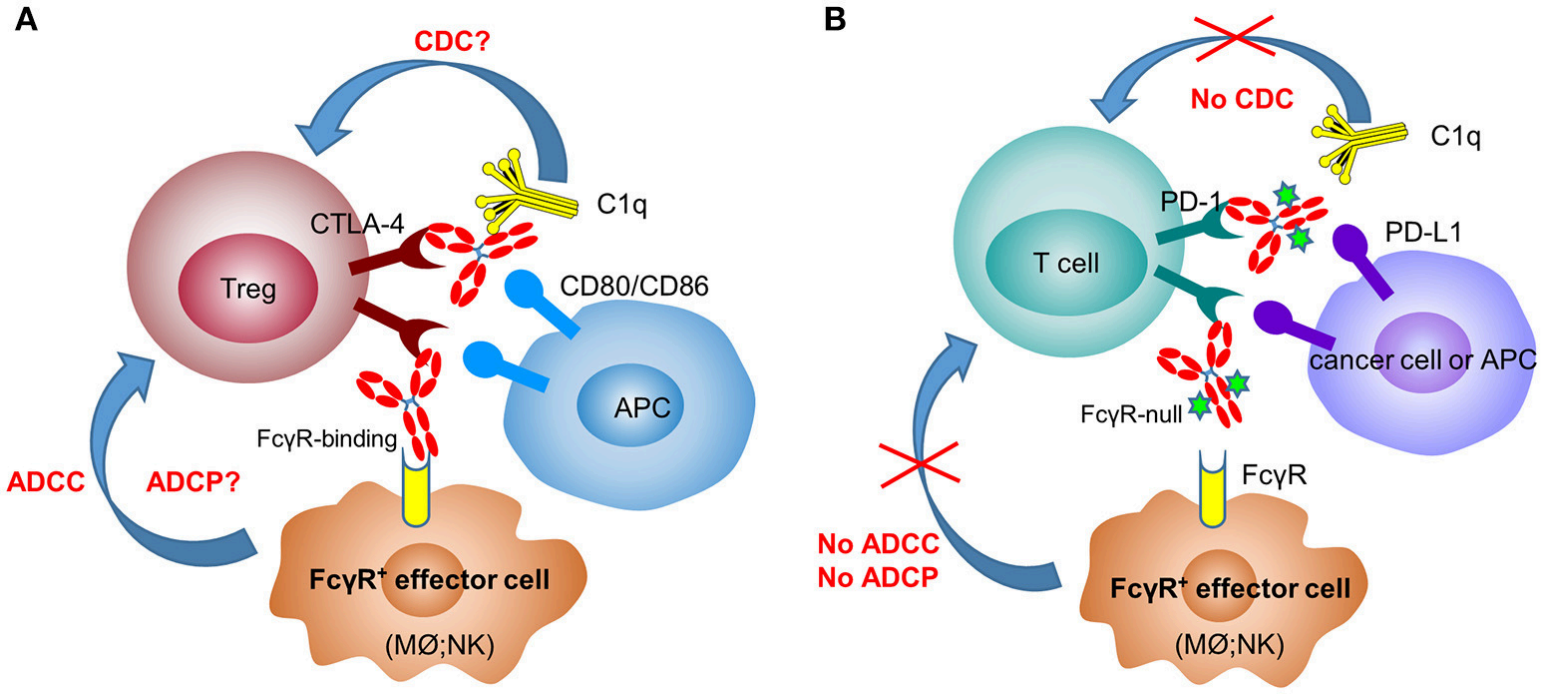
Anti-CTLA-4 and anti-PD-1 therapeutic antibodies have differential FcγR-binding requirement for optimal activity. In the mechanisms of action of anti-CTLA-4 antibodies **(A)**, depletion of Tregs after engaging FcγR^+^ effector cells [macrophages (Mϕ) and NK cells] plays a critical role in their efficacy. In contrast, anti-PD-1 antibodies need to have the Fc-mediated effector functions (ADCC, ADCP, and CDC) removed to avoid the killing of PD-1^+^ T cells by FcγR^+^ effector cells **(B)**.

In mouse tumor models (melanoma and colorectal cancer), several groups have clearly shown that surrogate anti-CTLA-4 antibody-mediated anti-tumor efficacy is dependent on Fc effector functions and correlate with depletion of tumor-infiltrating Tregs (10, 30, 56) (Figure 1A). In 2011, the FDA approved the first anti-CTLA-4 antibody, ipilimumab (IgG1 wild-type), for the treatment of melanoma. Furthermore, the combination of PD-1 blockade with ipilimumab demonstrated increased, durable anti-tumor activity in renal cell carcinoma and non-small cell lung cancer (NSCLC) (57, 58). Interestingly, anti-CTLA-4 clones, which lose the ability to block the B7-CTLA-4 interaction, remain fully active in inducing tumor rejection, suggesting that other mechanisms are involved in anti-CTLA-4 antibody-mediated anti-tumor efficacy besides the blocking of B7-CTLA-4 (59). In an *ex-vivo* assay, melanoma patient-derived non-classical monocytes could kill Tregs via ADCC (60). In addition, patients who responded to ipilimumab tended to have a higher percentage of CD14^+^CD16^+^ monocytes in the periphery. Using human FcγR-transgenic mice, Arce Vargas et al. clearly demonstrated that antibodies with isotypes equivalent to ipilimumab increased the CD8^+^ to Treg ratio by depleting intra-tumoral Tregs to promote tumor rejection (15). Furthermore, a response to ipilimumab in melanoma patients is associated with a high-affinity FcγRIIIa (CD16-V_158_) polymorphism. A second anti-CTLA-4 mAb, tremelimumab, is a human IgG2 isotype with minimal FcγRIIIa-mediated ADCC effects (61). However, anti-mouse CTLA-4 antibody with human IgG2 isotype could also deplete Tregs in human FcγR-transgenic mice in a FcγRIIa-dependent manner (15). Despite the convincing data from mouse models, there has not been direct evidence indicating that anti-CTLA-4 immunotherapy could efficiently deplete Tregs in human cancers (62, 63).

### PD-1/PD-L1

In recent years, immune therapy targeting the PD-1/PD-L1 pathway has become a backbone clinical strategy for cancer treatment. Programmed cell death 1 (PD-1) is an inhibitory immune modulatory receptor (64–66). It is inducibly expressed on activated T, NK, and B lymphocytes (67), macrophages, DCs (68), and monocytes (69) as an immune suppressor for both adaptive and innate immune responses. PD-1 is highly expressed on tumor-specific T cells. Engagement of PD-1 by its ligands, PD-L1 (70) or PD-L2 (71, 72) leads to the exhaustion of T cell function and immune tolerance in the tumor microenvironment. Blockade of PD-1 pathway has been shown to restore the function of “exhausted” T cells, resulting in significant anti-tumor activity (70, 73). To date, five PD-1 antibodies have been approved and many others are under development for the treatment of a broad spectrum of cancers (Table 2). Most of these anti-PD-1 antibodies are of IgG4 isotype with the S_228_P mutation (IgG4 S_228_P), which has similar effector-binding properties as the natural IgG4 with reduced ADCC and “null” CDC, but still retaining high affinity to FcγRI and binding to FcγRIIb. In the MC38 mouse model, Dahan et al. reported that engagement of FcγRs reduced the anti-tumor activity of an anti-PD-1 antibody by eliminating CD8^+^ tumor-infiltrating lymphocytes (TILs) via ADCC in a FcγRI-dependent manner (9). In addition, engagement of FcγRIIb by an anti-PD-1 antibody could also decrease its anti-tumor activities. Arlauckas et al. demonstrated that anti-PD-1 antibodies can be captured from the T-cell surface by FcγR-bearing macrophages. The blockade of FcγRs could thus prolong the binding of the anti-PD-1 antibody to CD8^+^ TILs and enhance the anti-tumor activity *in vivo* (74). A preclinical study by our group also suggested that FcγRI binding had a negative impact on the anti-tumor activity of anti-PD-1 antibodies in a humanized xenograft model. The binding could induce FcγRI^+^ macrophages to phagocytose PD-1^+^ T cells via ADCP and reverse the function of an anti-PD-1 antibody from blocking to activating (37). Recently, several published research papers documented the phenomenon that the hyperprogression frequencies of certain cancer types treated with FDA-approved anti-PD-1 antibodies were substantially higher than the control chemotherapy group (75–77). Lo Russo et al. linked the interaction between the anti-PD-1 antibody and FcγR^+^ macrophages to the hyperprogression in NSCLC during PD-1 blockade therapy (78). Based on these observations, an anti-PD-1 antibody with pure blocking activity would be more desirable, since an anti-PD-1 antibody with FcγR-binding activity can mediate cross-linking between PD-1^+^ T-cells and FcγR^+^ macrophages, induce the depletion of PD-1^+^ T effector cells, and thus compromise the T-cell activity of tumor growth inhibition (9, 37, 74) (Figure 1B).

**Table 2:** Select PD-1 and PD-L1 antibodies under development for cancer treatment.

**Target**	**Company**	**mAb**	**Clinical stages**	**IgG isotype or mutant with effector function nullified**
PD-1	Bristol-Myers Squibb	Nivolumab	Approved	IgG4 S_228_P
PD-1	Merck	Pembrolizumab	Approved	IgG4 S_228_P
PD-1	Regeneron/Sanofi	Cemiplimab	Approved	IgG4 S_228_P
PD-1	Novartis	Spartalizumab	Phase 3	IgG4 S_228_P
PD-1	BeiGene	Tislelizumab	Phase 3	IgG4mut, FcγR null
PD-1	Junshi	JS001	Approved	IgG4 S_228_P
PD-1	Hengrui	Camrelizumab	Phase 3	IgG4 S_228_P
PD-1	Innovent	Sintilimab	Approved	IgG4 S_228_P
PD-L1	Roche	Atezolizumab	Approved	IgG1mut, FcγR null
PD-L1	AstraZeneca	Durvalumab	Approved	IgG1mut, FcγR null
PD-L1	Merck KGaA/Pfizer	Avelumab	Approved	IgG1

Programmed death ligand 1 (PD-L1) is constitutively expressed by immune cells of myeloid lineages (79) and the cells at immune-privileged sites (80, 81). It is also inducibly expressed on T, NK and B lymphocytes, epithelial and endothelial cells upon stimulation by pro-inflammatory factors, such as IFN-γ and TNF-α (82). PD-L1 is the main ligand of PD-1, and the PD-L1/PD-1 axis is the major controller of the peripheral immune tolerance (65). In tumors, PD-L1 is expressed on both tumor cells (83) and tumor-infiltrating immune cells and can suppress anti-tumor immunity independently (84). Unlike anti-PD-1 antibodies, the three approved PD-L1 antibodies have differentiated FcγR-binding properties (Table 2). Atezolizumab and durvalumab are designed to eliminate FcγR-binding and effector functions (85, 86), while avelumab retains intact Fc functions (87). Recent preclinical data suggested that the engagement of FcγRs could augment the anti-tumor activity of anti-PD-L1 antibodies via the ADCC effect against the PD-L1^+^ immune suppressive myeloid cells (88) or tumor cells (89). However, it is also speculated that the effector function could be detrimental to the anti-tumor immunity due to the depletion of PD-L1^+^ APC cells and T effector cells. To understand the role of FcγR-binding on anti-PD-L1 anti-tumor efficacy, future studies are needed to elucidate the expression of PD-L1 in the tumor microenvironment and the effect of anti-PD-L1 antibody treatment.

### TIM-3

TIM-3 (T cell immunoglobulin and mucin-domain containing-3, also known as HAVCR2) is a member of the T-cell immunoglobulin- and mucin-domain-containing family that plays an important role in promoting T-cell exhaustion in both chronic viral infections and tumor escape from immune surveillance (90, 91). It is primarily expressed on immune cells, such as T cells, NK cells, DCs, and monocytes/macrophages (92). When expressed on effector T cells, activation of TIM-3 has been shown to reduce cytokine production, T-cell proliferation, and cytotoxicity, all of which could be rescued by TIM-3 blocking antibodies (93, 94). TIM-3 is also expressed on FoxP3^+^ Treg cells, especially in human tumor tissues, and is correlated with poor clinical parameters (95, 96).

Four TIM-3 ligands have been identified, which include PtdSer, Gal-9, carcinoembryonic antigen-related cell adhesion molecule 1, and high mobility group box 1 (97). To date, the detailed mechanisms of TIM-3 signaling remain unclear. Upregulation of TIM-3 expression in TILs, macrophages, and tumor cells has been reported in many types of cancers (98–101). Increased expression of TIM-3 in those cancers is associated with a poor prognosis and/or patient survival.

Following PD-1 antibody blockade, TIM-3 expression has been shown to be upregulated on TILs from both patient samples and animal models, resulting in “adaptive resistance” to anti-PD-1 treatment (102–104). Blockade of the TIM-3 receptor alone or in combination with PD-1/PD-L1 blockade has been shown both *in vitro* and *in vivo* to rescue functionally “exhausted” T cells (3, 93, 105).

In pre-clinical mouse models of colorectal cancer (MC38 and CT26), the effects of “silent” Fc vs. “competent” Fc on TIM-3 antibody-mediated anti-tumor activity with or without anti-PD-1 antibody treatment were evaluated by several groups (106, 107). The results showed that the combination of “Fc-silent” TIM-3 Ab with PD-1 Ab led to significantly more synergistic tumor-inhibitory effects than the one with “competent” Fc, while TIM-3 blocking Ab monotherapy demonstrated marginal anti-tumor efficacy. The exact mechanisms of Fc effector functions (ADCC and/or ADCP) in the negative regulation of anti-TIM-3 antibody-mediated anti-tumor efficacy remain unknown.

To date, the first-in-human phase 1/2 clinical trials have been initiated for four anti-TIM-3 antibodies: TSR-022 (NCT02817633), MBG543 (NCT02608268), BMS-986258 (NCT03446040), and LY3321367 (NCT03099109). TESARO recently released the clinical data of TSR-022, in monotherapy or in combination with an anti-PD-1 antibody (TSR-042) in patients who progressed following anti-PD-1 treatment (108). The results showed that the combination of TSR-022 and TSR-042 (500 mg) was generally well-tolerated in both NSCLC and melanoma patients, and clinical activities have been observed in the combination therapy, especially at a high dose of TSR-022 (300 mg) with an objective response rate (ORR) of 15% (3/20) and 40% stable disease (8/20) (108).

### LAG-3

LAG-3 (Lymphocyte activation gene-3, or CD223) is a member of the immunoglobulin superfamily (IgSF) (109). The immune-regulatory roles of LAG-3 were demonstrated in LAG-3 knockout mice, in which increased susceptibility to autoimmune diseases was observed (110, 111). LAG-3 is primarily expressed on activated T, natural killer (NK), and plasmacytoid dendritic cells (pDC), but not on resting T cells (109, 112). In addition, LAG-3 expression on Tregs is positively correlated with their immune-suppressive activity (113). Sequence homology analysis revealed that LAG-3 is structurally related to CD4, but with higher affinity (60 nM) to MHC class II (MHC-II) molecules, thus inhibiting CD4-MHC-II interaction and negatively regulating T-cell receptor (TCR) signaling (109, 114). In addition, LAG-3 can exert negative regulation of CD8^+^ T cells via CD4^+^ T cell-dependent and/or independent manners (115, 116). Similar to PD-1, LAG-3 is expressed on tumor-infiltrating lymphocytes (TILs), but to a less extent. Besides MHC-II molecules, LAG-3 has been shown to bind to galectin-3 (Gal-3) and LSECtin (115, 117). The exact biological function of these two ligands binding to LAG-3 remains unknown. Recently, fibrinogen-like protein 1 (FGL1) has been identified as a novel high-affinity ligand for LAG-3 (118). *In vitro*, FGL1 could induce T-cell inhibition in a LAG-3-dependent manner. In the MC38 colorectal cancer model, ablation of FGL1-LAG-3 interaction with either anti-FGL1 or anti-LAG-3 blocking antibodies inhibits tumor growth.

In mouse tumor models (Sa1N fibrosarcoma, MC38 colorectal cancer, and MBT-2 bladder cancer), dual blockade of LAG-3 and PD-1 receptors with blocking antibodies has shown to significantly improve the anti-tumor activity than either antibody alone (111, 119). In a study by Jun et al., a pair of anti-mouse LAG-3 surrogate antibodies with IgG1 (D265A) [anti-mLAG-3 IgG1(D265A)] or IgG2a (anti-mLAG-3 IgG2a) isotypes were generated based on a commercial clone (C9B7W). Comparative study of these two antibodies either alone or in combination with anti-mouse PD-1 antibody in the CT26 mouse colorectal cancer model showed that anti-mouse LAG-3 antibody with minimal Fc effector functions [IgG1 (D265A)] had anti-tumor efficacy, and the one with effector function (IgG2a) had no apparent tumor inhibitory effect (120). In addition, when combined with PD-1 blocking antibody, anti-mLAG-3 IgG1 (D265A) showed significantly synergistic anti-tumor effects, whereas anti-mLAG-3 IgG2a with intact effector function in combination with an anti-mouse PD-1 antibody was less efficacious than anti-mouse PD-1 alone, suggesting that the effector function of LAG-3 antibody might interfere with anti-mouse PD-1 mediated efficacy. The anti-tumor efficacy of anti-mouse LAG-3 antibodies without effector functions was also observed by other groups (119, 121, 122).

As of now, there are six LAG-3 antibodies being evaluated in clinical trials. All these LAG-3 antibodies have Fc with either reduced or “null” effector functions. Preliminary data showed that combining anti-LAG-3 therapy (BMS-986016) with nivolumab in melanoma patients refractory to PD-1/PD-L1 treatment could help patients overcome resistance and restore T-cell function with an ORR up to 18%, especially in patients with high LAG-3 expression (≥1%)(123).

### TIGIT

TIGIT (T cell immunoglobulin and ITIM domain, also known as WUCAM or Vstm3) is a member of the CD28 family of proteins that play an important role in inhibiting T- and NK cell-mediated functional activities in anti-tumor immunity (124–126). TIGIT is mainly expressed on T and NK cells. T cells in the tumor microenvironment (3) often co-express TIGIT with other “checkpoint” inhibitory immune receptors, such as PD-1, LAG-3, and TIM-3 (93, 127).

Two TIGIT ligands, CD155 (PVR) and CD112 (PVRL2, nectin-2), have been identified; they are primarily expressed on APCs (such as dendritic cells and macrophages) and tumor cells (125, 126, 128, 129). The binding affinity of TIGIT to CD155 (*Kd*: ~1nM) is much higher than to CD112. Whether the TIGIT: CD112 interaction is functionally relevant in mediating inhibitory signals is yet to be determined. High-affinity binding of TIGIT to CD155 could compete with another co-stimulatory receptor, CD226 (DNAM-1), which binds to the same ligands with lower affinity (*Kd*: ~100nM) and delivers a positive signal (130), therefore reducing T- or NK-activation. In addition, the interaction between TIGIT and PVR on dendritic cells (DCs) could deliver a “reverse signaling” in DCs, leading to reduced DC activity and T-cell activation (126). TIGIT expression on Tregs has been associated with a highly immune-suppressive phenotype in tumor tissue and TIGIT signaling in Tregs may favor Treg stability (131, 132).

Blockade of the TIGIT receptor alone or in combination with PD-1/PD-L1 blockade could rescue functionally “exhausted” T cells both *in vitro* and *in vivo* (133, 134). In the CT26 cancer model, Fc with effector functions is critical for TIGIT antibody-mediated anti-tumor activity (11, 135). The TIGIT antibody with wild-type (WT) human IgG1 Fc (EOS884448) has been shown to be capable of preferentially depleting Treg cells *in vitro* (11). The authors demonstrated that the surrogate mouse TIGIT antibody of the mIgG2a isotype has potent anti-tumor activity either as monotherapy or in combination with a PD-1 antibody. In contrast, the mouse anti-TIGIT antibody with Fc devoid of effector functions did not show any of the anti-tumor efficacies, indicating that Fc-mediated effector functions are required for TIGIT antibody-mediated anti-tumor effects. In addition, the observed efficacy was associated with increased activity of effector T cells (CD8^+^ and CD4^+^) and also with Treg depletion within the TME. Argast et al. also observed that effector functions were critical for TIGIT antibody-induced *in vivo* efficacy (135).

To date, there are six TIGIT antibodies (see Table 3) in clinical trials, with different IgG isotypes or mutant forms. The most advanced, MTIG7192 (NCT03563716), is in a phase 2 trial in combination with the anti-PD-L1 antibody atezolizumab for treatment of advanced NSCLC. How the effector functions affect clinical activities remains to be seen.

**Table 3:** Anti-TIGIT in clinical trials.

**Company**	**mAb**	**Clinical stages**	**IgG Isotype and Fc effector functions**
Genentech	MTIG7192	Phase 2	IgG1
Merck Sharp & Dohme	MK-7684	Phase 2	IgG1
Bristol-Myers Squibb	BMS-986207	Phase 1/2	IgG1mut, FcγR null
Oncomed	OMP-313M32	Phase 1	IgG1
Arcus	AB-154	Phase 1	IgG4 S_228_P
Potenza	ASP8374	Phase 1	IgG1mut, FcγR null

### CD73

CD73 (also known as 5'-ecto-nucleotidase, or NT5E) is a glycosylphosphatidylinositol (136) anchored cell surface protein, which has both enzymatic and non-enzymatic functions (137). As a nucleotidase, it catalyzes the extracellular dephosphorylation of adenosine monophosphate (AMP) to adenosine. Adenosine is believed to be an immunosuppressive molecule inhibiting CD8^+^ T cells, NK cells, and dendritic cells, while promoting the proliferation of immunosuppressive cells (138, 139). In some cases, CD73 can be shed from the cell surface with retained enzymatic activity (140). Expression of CD73 varies on normal tissues but remains at constitutively high levels on many types of cancer cells. High CD73 expression has been shown to be correlated with unfavorable clinical outcomes (141–147), which is consistent with the immunosuppressive role of adenosine.

Three CD73 blocking antibodies have been entered into clinical trials (i.e., BMS-986179, CPI-006, and MEDI9447). Compared with small-molecule inhibitors, anti-CD73 mAbs offer the possibility of directly targeting both enzymatic and non-enzymatic CD73 pathways (148). *In vitro* data showed that MEDI9447 (human IgG1 variant) could inhibit the enzymatic activity of both soluble- and membrane-bound CD73 through prevention of the conformational transition of CD73 to an active state, and could induce internalization of membrane-bound CD73, and restore T-cell proliferation from the inhibition by AMP (149, 150). In a mouse model, MEDI9447 monotherapy showed significant anti-tumor efficacy, which was further increased when combined with a PD-1 antibody (150). In the Fc region of MEDI9447, triple mutations (L_234_F/L_235_E/P_331_S) were introduced to eliminate its binding to FcγRs (Including FcγRI, FcγRIIa, and FcγRIIIa) and C1q (150, 151). Similarly, CPI-006 from Corvus is also an IgG1 isotype with a “silent” Fc. It could fully block the production of adenosine by inhibiting the enzymatic activity of CD73 (IC50, 17nM) without internalization, while also activate B cells independent of adenosine reduction (152).

Another anti-CD73 antibody, BMS-986179, is an IgG2/IgG1 hybrid with a “null” effector function. BMS-986179 could not only inhibit CD73 enzymatic function but also induce rapid, near-complete internalization (153). The disulfide bond isomerization of IgG2 is thought to be the major mechanism for BMS-986179-induced CD73 efficient clustering and internalization. Results from mouse models indicated that the combination of PD-1 blockade and a surrogate anti-mouse-CD73 antibody treatment resulted in more enhanced anti-tumor efficacy than either treatment alone (153). In a phase 1/2a study (NCT02754141), 59 patients with advanced solid tumors were treated either alone with BMS-986179 or in combination with nivolumab. Preliminary results showed that both the monotherapy of BMS-986179 and the combination were well-tolerated and clinical activities were observed with 7 partial responses and 10 stable diseases (154).

### VISTA

VISTA (V-domain Ig-containing Suppressor of T cell Activation, also known as B7-H5, B7H5, C10orf54, DD1alpha, GI24, PD-1H, PP2135, SISP1) is a type I transmembrane protein with a single extracellular IgV domain, functioning as a negative regulator of T-cell immunity. It is predominantly expressed on hematopoietic cells, at the highest level on myeloid cells and at lower levels on T cells (155). *In vitro* studies indicated that not only could VISTA-Ig inhibit T-cell activation and proliferation, but it could also induce Treg differentiation (155). The receptor for VISTA remains unknown. Results from murine models suggested that VISTA and PD-1 suppressed T-cell function in a synergistic manner, providing the possibility of combined therapy targeting both VISTA and PD-1 to enhance anti-tumor immunity (156).

To date, JNJ-61610588, a fully human IgG1 antibody (with wild-type Fc) is the only anti-VISTA monoclonal antibody in a clinical trial (NCT02671955). A preliminary study showed that JNJ-61610588 could induce monocytes and T-cell activation, as well as T-cell proliferation *in vitro* (12). Interestingly, active Fc and Fc receptor crosslinking is required for the efficacy, since neither the silent Fc version of VSTB140, with an IgG2 sigma constant region, nor the Fc blocking of JNJ-61610588 exhibited activity. Consistent with *in vitro* findings, the anti-tumor activity of JNJ-61610588 in mouse tumor models was observed. The exact mechanisms and clinical evidence remain to be seen.

### B7-H3

B7-H3 (Human B7 homolog 3, also known as CD276) is a member of the B7 family of immune proteins. The majority of studies suggest that B7-H3 is an immune checkpoint molecule (157–159), although it was initially characterized as a co-stimulatory molecule for T-cell activation and IFNγ production (160). The B7-H3 receptor expressed on T cells remains to be identified (161). B7-H3 has limited expression on normal tissues but is preferentially expressed on a wide spectrum of cancer cells and tumor vasculature, which is associated with poor outcomes in multiple cancers (162–168).

MGA271 (or enoblituzumab), is an Fc-enhanced humanized IgG1 anti-B7-H3 antibody developed by MacroGenics. Mutations were introduced in the IgG1 Fc domain to increase its affinity to FcγRIIIa but decrease the affinity to FcγRIIb (169). Enhanced ADCC against a wide arrange of B7-H3 positive tumor cell lines (including prostate, lung, breast, colon, bladder, renal cancers and melanoma) was observed across all the donors with different FcγRIIIa polymorphisms (low-affinity 158F homozygous, high-affinity 158V homozygous, and 158F/V heterozygous). Consistent with *in vitro* data, greater anti-tumor efficacy was observed in the group with MGA271 than the one with wildtype IgG1 Fc in human FcγRIIIa-158F-transgenic mice (170). Initial evidence of anti-tumor activity was observed in a clinical trial with MGA271, with no dose-limiting toxicities or severe immune-related side effects (171).

## Concluding Remarks

In this review, we have summarized recent advances in the study of FcγR-binding on checkpoint antibody therapy. For targets such as CTLA-4, multiple studies indicated the critical role of competent IgG1-Fc for anti-CTLA-4 antibody-mediated intratumoral depletion of Tregs *via* ADCC (10, 15). This MOA may largely be attributed to the preferential surface expression of CTLA-4 on Tregs and the presence of significant numbers of CD16^+^ macrophages inside tumors (15). In mouse models, anti-CTLA-4 mAbs do not block CTLA-4-B7 interaction, yet they remain active in anti-tumor efficacy, suggesting that intratumoral depletion of Tregs by anti-CTLA-4 antibodies might be the primary MOA (172). A similar phenomenon was observed for TIGIT or VISTA in mouse models, in which their antibody-elicited anti-tumor efficacy is mainly dependent on Fc-mediated effector functions (11).

So far, five approved anti-PD-1 mAbs (nivolumab, pembrolizumab, and cemiplimab) are of human IgG4 isotype. The choice was made primarily based on the fact that the affinity of IgG4 to FcγRIIIa is very low, inducing little ADCC (13). However, IgG4 binds to FcγRI with high affinity, which can negatively impact the efficacy of PD-1 therapy (9, 37). Moreover, IgG4 can also bind to FcγRIIb, leading to reduced anti-tumor efficacy, likely through the induction of a more immunosuppressive environment (9, 78). Therefore, an IgG variant of the anti-PD-1 antibody with null FcγR-binding is expected to be the optimal candidate for therapeutic blocking of PD-1 without the unwanted engagement of FcγR pathways. A similar rationale applies to co-inhibitory receptors TIM-3 and LAG-3, in which blocking antibody-mediated anti-tumor efficacy might be compromised when the Fc maintains intact effector functions.

Three PD-L1-targeting mAbs have been approved: atezolizumab, durvalumab (IgG1 variant with null or reduced Fc-FcγR binding), and avelumab (wild-type IgG1, ADCC-enabling) (173). Comparison of clinical activities of these mAbs may provide important insight into the contribution of FcγRs for the anti-PD-L1 treatment of human cancers.

It should be noted that most of the findings in this review about the role of IgG antibody and FcγR binding on immune-oncology therapy were obtained from mouse models (some even in human FcγR-transgenic mice). There are several factors that need to be taken into consideration, including, how well the mouse FcγR expression pattern (including transgenic human FcγRs) mimics the human counterpart, especially in cancer patients, and how different the abundance and distribution of FcγR^+^ effector cells (e.g., NK cells and macrophages) are in mice vs. in humans in the TME. Studies on the impact of human FcγR polymorphisms (FcγRIIIa-V_158_ vs. F_158_; FcγRIIa-H_131_ vs. R_131_) on clinical activity may also shed light on the MOA of immune checkpoint-targeted antibodies (15). In addition, *ex vivo* assays using human tumor samples and targeted antibodies in various settings may provide useful insight into this matter.

In summary, the triggering of effector functions on IgG and FcγR interactions is a complex process; the overall outcome may be dependent on the target expression level, distribution, and abundance of T cells, and the FcγR^+^ effector cells (NK cells and macrophages) inside tumors. Further investigation through clinical pathology and pharmacology studies is needed to assess the translational applicability of these findings in mouse models to human cancer treatment.

## Author Contributions

All authors listed have made a substantial, direct and intellectual contribution to the work, and approved it for publication.

### Conflict of Interest Statement

This work was funded by BeiGene. All authors have an ownership interest in BeiGene.
